# Optimization of the Multienzyme-Assisted Extraction Procedure of Bioactive Compounds Extracts from Common Buckwheat (*Fagopyrum esculentum* M.) and Evaluation of Obtained Extracts

**DOI:** 10.3390/plants10122567

**Published:** 2021-11-24

**Authors:** Paulina Štreimikytė, Dalia Urbonavičienė, Aistė Balčiūnaitienė, Pranas Viškelis, Jonas Viškelis

**Affiliations:** Lithuanian Research Centre for Agriculture and Forestry, Institute of Horticulture, 54333 Babtai, Lithuania; dalia.urbonaviciene@lammc.lt (D.U.); aiste.balciunaitiene@lammc.lt (A.B.); pranas.viskelis@lammc.lt (P.V.); jonas.viskelis@lammc.lt (J.V.)

**Keywords:** *Fagopyrum esculentum*, enzyme-assisted water extraction, optimization, hydrophilicity, non-starch polysaccharide enzymes, *Trichoderma reesei*

## Abstract

Optimization of the extraction procedure using a multienzymes cocktail for common buckwheat (*Fagopyrum esculentum* M.) is important due to the yield, fermentable sugars, oligosaccharides and bioactive compounds for creating higher added value products. This study was undertaken to find out the optimum multienzymes-water extraction on yield and total phenolic compounds for common Buckwheat using response surface methodology (RSM). Three independent variables, time (2, 13, and 24 h), temperature (60 °C, 70 °C, 80 °C), and non-starch polysaccharide (NSP) enzymes mixture (0.10, 0.55, and 1.00 mL), were analyzed to optimize the response variables. NSP hydrolyzing enzymes, cellulase, xylanase, and β-glucanase, were produced by *Trichoderma reesei*. Estimated optimum conditions for *F. esculentum* were found: time—2 h, temperature—65 °C, and cellulase activity—8.6 CellG5 Units/mL. Different optimization run samples were collected and lyophilized for further analysis until the hydrophilic property using the water contact angle methodology and rutin content using HPLC was determined. Results indicated NSP enzymes activity did not differ between water contact angles after 13 h of enzymatic water extraction. However, longer fermentation time (24 h) decreased static water contact angle by approximately 3–7° for lyophilized water extract and 2–7° for solid fraction after fermentation. It implies enzymatic hydrolysis during water extraction increased hydrophilic properties in solid fraction and decreased hydrophilicity in water fraction due to the enzymes cleaved glycosidic bonds releasing water-soluble compounds.

## 1. Introduction

Common buckwheat (*Fagopyrum esculentum* M.) are well-known grains for their high nutritional and functional value. They are rich in flavonoid content, especially rutin, which is able to cause antioxidant activity, enhance technological properties, e.g., foaming, and are widely approached in many fields, including nutraceutical and food industries [1,2].

Recently, rising demand for higher nutritional value, plant-based trends and the ‘clean label’ trend increased the bioactive compounds needed in food [3]. Moreover, phenolic compounds found in plant tissues are getting more attention not just for their health-promoting benefits [4,5]. These substances are used as natural food additives due to their antioxidant and antimicrobial properties [6,7,8,9]. 

Nevertheless, phenols are found in free and bound forms [10]. Studies show that common buckwheat phenolic compounds such as chlorogenic acid, myricetin, quercetin, and rutin are in bound form. These compounds are determined in anti-inflammatory, anti-cancer, cardioprotective, and anti-allergic properties [11]. Extraction of these substances requires organic and often toxic chemicals [12]. However, enzymes in extraction methods play a tremendous role as an innovative and environmentally friendly enchanter of biochemical compound extraction. 

Rutin (quercetin-3-rutinoside) is a secondary metabolite found in the majority of plant groups; however, for cereal and pseudo-cereal categories it is only found in buckwheat’s [1,13]. In plants, the function of rutin is to protect it from harmful ultraviolet radiation and diseases [14]. Depending on the morphological part, the technological process of buckwheat flours, rutin content varies from 50 to 200 mg/kg [13]. Although, many studies show significant rutin applications in the food and pharmaceutical industries to improve human health [15]. Zapata-Morales et al. [16] approached rutin-paracetamol and rutin-NSAIDs combination studies, which determined that the synergistic activity is a more efficient anti-inflammatory drug [1]. Recent studies also determined the technological properties of rutin and soy protein interaction due to stabilized emulsions and foam formation [17,18,19]. 

In the food industry, enzymatic hydrolysis creates syrups, plant-based beverages and increases bound phenolics in extracts [16,20,21]. Carbohydrases are potently used for grain materials and can be diversified to the amylolytic and cellulolytic enzymes and are typical for saccharification for grains due to increased fermentable sugars [22,23]. Enzymes hydrolyze starches, cellulose, hemicellulose, lignin, and pectin, releasing proteins and phenolic compounds [10]. In general, the enzymatic hydrolysis of the cereal matrix leads to the degradation of antinutrients, which increase the nutritional value and availability of phenolic compounds, proteins, and carbohydrates [24]. Released substances, usually powdered, are also well incorporated in the food production chain for sensory, technological, and rheological properties without additives [25]. However, different polarities indicate the hydrophilicity of enzymatically hydrolyzed powdered forms, which impact media dispersity, critical for homogeneous fusion [2]. For this reason, thermodynamic surface properties, such as contact angle, are exciting tools for dispersity determination in powdered forms [26,27]. 

Furthermore, it is known that multienzyme extraction is more efficient than mono-enzyme extraction [28,29]. For this reason, in industries, non-starch polysaccharides enzymes are usually used where cocktail composition of different types of enzymes varies, including cellulases, xylanases, and others [30]. The type of cereal and its biochemical composition play a leading role in enzymatic hydrolysis due to the different enzymes/substrate ratio, temperature, and pH [25,31]. Specific enzymes release different compounds from plant cells, but there are missing data about the effect of multienzyme hydrolysis during different extraction methods, parameters, phenolic compound release, yield, and the impact of hydrophilicity.

This study was undertaken to find out the optimal parameters of multienzymes-water extraction on yield, total phenolic compounds, and rutin variation for *Fagopyrum esculentum* using response surface methodology (RSM). Furthermore, the hydrophilicity was tested using water contact angle methodology.

The hypothesis of the study is that different amounts of non-starch polysaccharide enzymes in the variation of parameters during extraction release compounds in common buckwheat differently.

## 2. Results and Discussion

### 2.1. Optimization of Multienzyme-Assisted Extraction

The main aim of applying hot water multienzyme-assisted extraction for common buckwheat was to optimize this process for the isolation of hydrophilic substances. The main advantages of water extraction using a multienzymes mixture is its cost-effectiveness, environmental friendliness and it releases insoluble bounds of phenols into more bioactive parts [14]. The adequacy of the model can be prescribed from the determination coefficient R-Squared of 0.8560, which indicates a good fit of the model to the experimental data. Additionally, the predicted R-Squared of 0.7033 is in reasonable agreement with the adjusted R-Squared value of 0.8046. Adequate precision indicated an adequate signal, which indicates that model can be used to navigate the design space. Moreover, this model can be considered reasonably reproducible as indicated by the coefficient of variation (CV), 3.54%.

**Figure 1:**
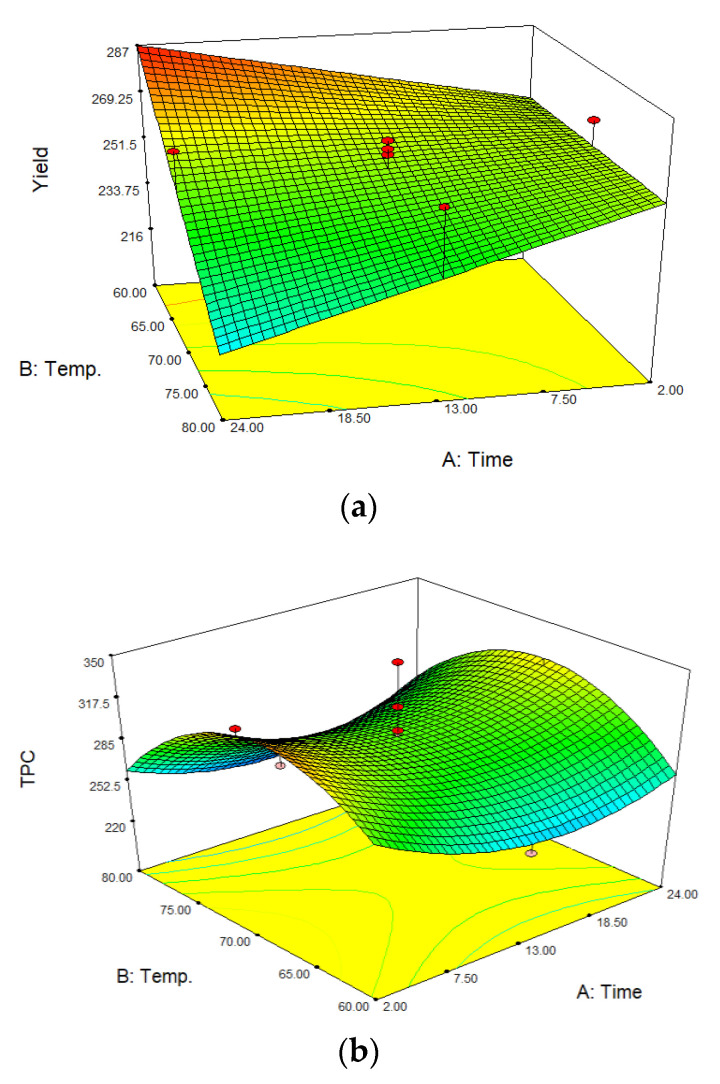

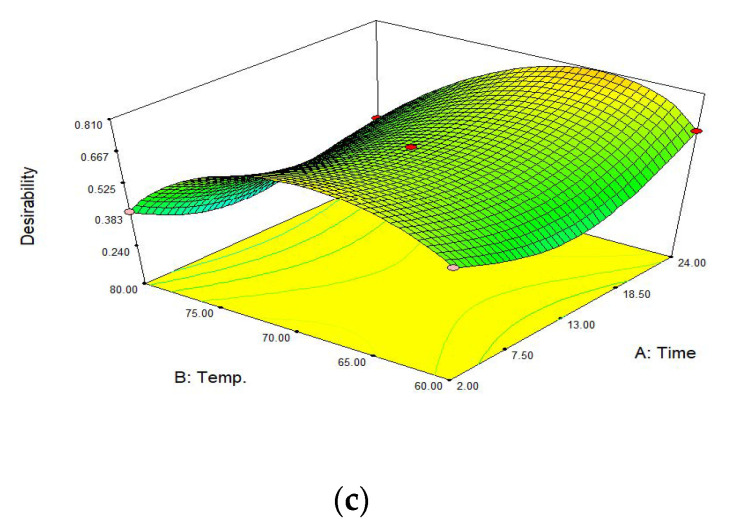
RSM plots of the dependencies of buckwheat flour extract on yield, total phenolic content, and desirability. In plots, axes are named by time (hours), Temp. (temperature, °C), TPC (mg GAE/100 g), and yield (g). Where subfigures, (**a**)—effect of extraction yield and time; (**b**)—effect of extraction TPC and time; (**c**)—effect of extraction temperature and time.

Through multiple regression analysis of the experimental data, the model for predicted response Y identified as TPC and yield could be expressed with the following quadratic polynomial equation, presented in Figure 1.

(1) $$Y\left(TPC\right)=297.92-4.50X_{1}-9.40X_{2}+68.90X_{3}+8.88X_{1}X_{2}+11.63X_{1}X_{3}+4.63X_{2}X_{3}+28.45X_{1}{}^{2}-51.05X_{2}{}^{2}-1.05X_{3}{}^{2}$$

(2) $$Y\left(Yield\right)=223.68+10.66X_{1}-0.43X_{2}+31.30X_{3}-0.15X_{1}X_{2}+0.12X_{1}X_{3}-0.30X_{2}X_{3}$$

Equation (1). The model for the predicted response *Y* by the quadratic polynomial equations where $X_{1}$—time; $X_{2}$—temperature, and $X_{3}$—NSP

The effect of three variables on the extract yield and total phenolic content was selected. Extraction time (hours), temperature (°C), and non-starch polysaccharides enzyme mixture (NSP, mL) were chosen as independent variables, and three levels of each of them were set (Table 1). The total slurry mass in samples before the performed design run was approximately 330 g.

Experimentally obtained extract yields from buckwheat flours were from 196 to 289 g. Consequently, the water extracts’ total phenolic content was from 228 to 350 mg GAE/100 g. The desirability function was implemented to describe multiple responses. The optimal conditions obtaining the desirability 0.808 with the highest hydrophilic extract yield (272 g) and total phenol content (324 mg GAE/100 g) were 24 h, 65 °C, and cellulase activity of 8.6 CellG5 Units/mL. However, 17 solutions with desirability in the range of 0.808 to 0.747 were suggested, that is, ten solutions after 2 h of extraction and seven solutions after 24 h of extraction. The highest TPC (339 mg GAE/100 g) was obtained after 2 h, 68 °C, and cellulase activity of 2.58 CellG5 Units/mL. Temperature and pH are the critical factors in the activity and specificity of the enzyme to separate the substrates [31]. In the experimental design, pH was 6.6. Scientific approaches indicate that the optimum conditions for amylolytic enzymes (amylases and glucoamylases), are at the temperature of 68 °C and at a pH close to physiological conditions (around 7.0). This temperature increases their hydrolyzing properties [32,33]. Therefore, amylolytic enzymes release fermentable sugars, and observations from other authors’ results indicate that sugar increases significantly and stabilizes in the first few hours of fermentation. Although, fermentable sugars slightly decreased after 12 h [33]. These tendencies correlate with the results of TPC in experimental design. Consistently, for phenolic content, cellulolytic enzymes break down the plant cell walls and release bioactive molecules specifically. The manufacturer of the non-starch polysaccharides enzymes mixture declares that the optimal activity range is from 34 to 75 °C and pH in the range of 4.5–7.0. In these particular optimum conditions, the results correlate with the manufacturer’s. Moreover, enzyme assisted extraction is getting more attention for higher TPC content compared to ultrasound-assisted extraction (UAE) and high-pressure processing (HPP). In comparison, enzymatic hydrolysis in lemon flavedo demonstrated higher TPC release comparable to HPP and UAE, which indicates that enzymes promoted the release of bioactive compounds [34].

The yield after multienzyme-assisted extraction of buckwheat flour increased. The higher extraction temperature decreased the yield. Water evaporation is higher at 80 °C than 60 °C, which had an impact on yield. However, compared to the control sample yield, it is around 2 times higher from 113 ± 36 g to 255 ± 20 g, respectively. The amylolytic enzymes, such as enzyme α-amylase presents the properties of liquefying the starch acting in the internal α 1 → 4 linkages, resulting in the rapid loss of viscosity of the suspension [35]. Liquefied suspension creates the ability to disperse multienzymes in the matrix uniformly. These changes have an impact on the higher conjunction between enzyme and substrate. 

### 2.2. Determination of Rutin

**Figure 2:**
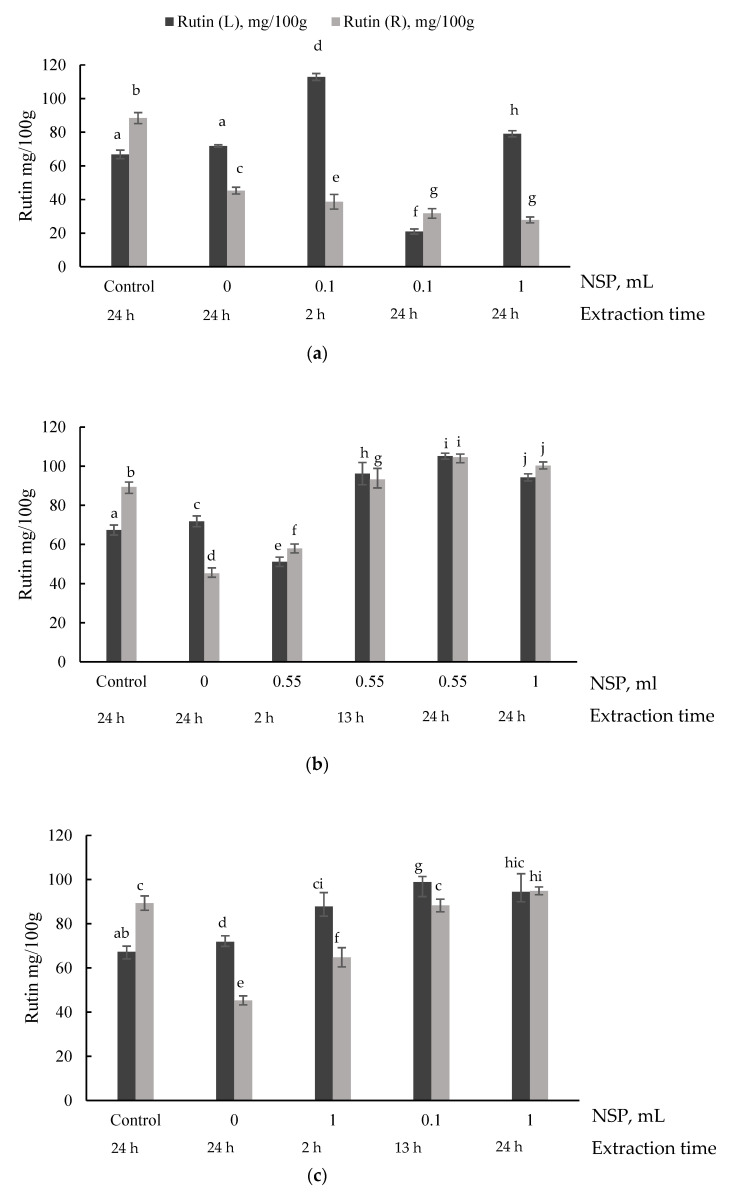
Rutin determination in different samples after optimization runs at: 60 °C temperature (**a**), 70 °C temperature (**b**), and 80 °C temperature (**c**). Where rutin (L) mg/100 g—rutin content in liquid phase, rutin (R) mg/100 g—rutin content in residues. X abscissa indicates a non-starch polysaccharides enzyme cocktail in the mixture and extraction time of each sample; 0 sample is with amylolytic enzymes without NSP and the control sample without enzymes after water extraction at room temperature; a–i letters for the same analytical parameter, above the columns, show a significant difference between the samples at the same temperature (*p* > 0.05).

The effect of the NSP enzymes cocktail quantity, temperature, and time on the contents of rutin in the common buckwheat liquid fraction and residues are characterized in Figure 2. Ethanol (70%), which is regarded as GRAS, was used to extract rutin from the *F.esculentum* lyophilized fractions after optimization runs [4]. Before the hydrothermal treatment, the control sample without enzymes cocktail showed the content of rutin for the liquid phase as 67.31 mg/100 g and residues of 89.34 mg/100 g. Previous studies approached the thermal stability of rutin, which reflects the results of the 70 °C and 80 °C treatment [4,36]. In general, complex treatment of buckwheat flours showed increased content of rutin. Even minimal dosages of NSP showed a significant impact of releasing rutin after 2 h in a range of 87.78–112.90 mg/100 g. Scientific approaches prove enzyme-assisted extractions enhance active biological compounds in extracts due to the specific cleavage of a complex matrix [16]. For example, in subcritical water extraction, rutin concentration after extraction varies from 60.20 to 70.50 mg/100 g [37]. Results indicate that rutin may occur between soluble and insoluble fibers likewise rhamnogalacturonan, homogalacturonans, xyloglucans, and mannans [29,38]. However, enzymes assistance is usually incorporated into multistep processes. Correspondingly, biorefining pomaces due to singular enzyme assisted extraction is less efficient for polar constituents than pressurized liquid extraction or supercritical carbon dioxide extraction [39,40]. 

### 2.3. Water Contact Angle

Twenty runs of optimization were performed, and three different lyophilized samples of the hydrophilic and solid fraction at 70 °C were collected to perform water contact angle (Table 2). The control sample was performed at 20 °C without any enzymes. Powders have a significant role in many applications such as pharmaceutical drugs and food products development. The technique is based on discussing the wetting tendency. Wettability of the powder is important when considering the processes and properties of the final products [27]. In general, all tested samples showed a water contact angle range from 35.7° to 57.5°, which describes samples as ‘wetting liquid’ that forms a contact angle with the solid due to the angle being smaller than 90° [2]. 

**Figure 3:**
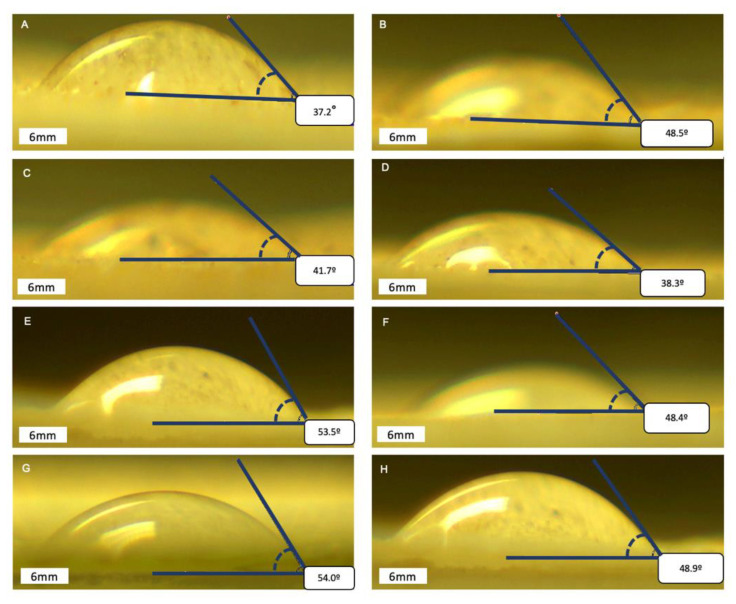
Water contact angle images in different samples after optimization runs, where **A**—control sample solid fraction; **B**—control sample water fraction; **C**—1 sample solid fraction; **D**—1 sample water fraction; **E**—2 sample solid fraction; **F**—2 sample water fraction; **G**—3 sample solid fraction; **H**—3 sample water fraction.

The measurement results represented in Table 2 and Figure 3 indicate that cellulase activity did not differ between water contact angles after 13 h of enzymatic water extraction. However, longer extraction time (24 h) decreased the static water contact angle by approximately 6.7 degrees for lyophilized hydrophilic extract and 2.3 degrees for solid fraction after fermentation. In general, multienzyme extraction decreased the water contact angle in the solid fraction and increased the water contact angle in the water fraction. It implies enzymatic hydrolysis during water extraction increased hydrophilic properties in the solid fraction and decreased hydrophilicity in water fraction. The enzymes’ cleaved glycosidic bonds released water soluble compounds. Additionally, a small amount of lipophilic substances were obtained in liquid fraction, which may impact hydrophilicity.

## 3. Materials and Methods

### 3.1. Plant Material (Dependent Sub-Sample)

Organic roasted common buckwheat flour (*F. esculentum* M.) materials were obtained from local Lithuanian producers (Producer “Ekofrisa”, Prienai, Lithuania). The nutritional declaration of the product is presented in Table 3. For the experiment, the supplier randomly took three different batches of 1 kg from the stock.

### 3.2. Enzyme Products (Independent Sub-Sample)

Grainzyme NL is a classical multienzyme product used as an adequate substitute for reducing the viscosity of grain products. The main enzyme mixture component is cellulase. The product is declared to have a 5000 U/mL activity of cellulase. Furthermore, the manufacturer states that the enzymes mixture contains various hemicellulose, endo-xylanase, egzo-xylanase, beta-glucanase, mannanase, galactosidase and pectinase activities. Enzymes were produced from *Thrichoderma Reseii* (Suntaq, Guanghzou, China). 

SQzyme AGL is a mono-component glucoamylase [EC3.2.1.3] derived from the fermentation of a wild type of *Aspergillus niger* (Suntaq, Guanghzou, China). The product is declared to have a glucoamylase activity of 150,000 U/mL.

### 3.3. Experimental Design of Multienzyme-Assisted Extraction Optimization and Statistical Data Handling

*F. esculentum* flour samples were extracted using hot water multienzymes-assisted extraction. Response surface methodology (RSM) using a central composite design (CCD) was used to determine the optimal multienzymes-water extraction on yield and total phenolic compounds for common buckwheat. For data analysis and established data, the software Design-Expert 7.0.0 (Stat-ease, Inc., Minneapolis, MN, USA) was used. Three independent variables, time (2, 13, and 24 h), temperature (70 °C, 80 °C, 90 °C), and non-starch polysaccharide (NSP) enzymes mixture (0.1 mL, 0.55 mL, and 1.00 mL), were analyzed to optimize the response of variables. The desirability function was implemented to describe and obtain an optimized multiple response of method. Variables of time, temperature, and NSP were chosen in the range. Yield and total phenolic content were chosen as maximized goals. The adequacy of the model was determined by evaluating the determination coefficient, adjusted R-squared and predicted R-squared.

The water and buckwheat flour ratio was 5:1. For the enzyme combinations Grainzyme NL and SQzyme AGL were used. The enzymatic activity of cellulase, glucoamylase, and α-amylase was determined in accordance with the protocol K-CERA 06/18 and KCellG5-4V 06/18 (Megazyme, 2018). Furthermore, the estimated activity of cellulase, glucoamylase, and α-amylase was 2300 CellG5 Units, 150,000 U/mL, 18,000 U/mL, respectively. Afterwards, amylolytic enzymes mixture was added to reach the enzyme/substrate ratio of 0.5% *v*/*w*. Enzyme-assisted water extraction tests were carried out at 70–80 °C in an incubator. After extraction, the hydrolysis was terminated by heating the water up to 98 °C for 10 min. Separation of the slurry into liquid and solid fractions was carried out using a filter (200 mesh). Hydrophilic extract samples were taken from the received liquid hydrolysate. Pomace samples were taken from the solid hydrolysate. Continuously, samples were lyophilized and stored at room temperature. The extracts of liquid and solid hydrolysates obtained were consecutively re-extracted for further analysis. All the experiments were carried out in triplicate.

### 3.4. Determination of Total Phenolic Content (TPC)

TPC was assessed spectrophotometrically using a Folin–Ciocalteu reagent as described in the work [36]. The total phenolic content was determined by the equation (y = 10.56X + 0.0189, r^2^ = 0.997) of the calibration curve of gallic acid and expressed in mg/100 g, the equivalent of gallic acid for the dry raw material. The absorbance was measured using a Genesys-10 UV/Vis spectrophotometer (Thermo Spectronic, Rochester, NY, USA), at 765 nm wavelength. Lyophilised samples after multienzymes water extraction were re-extracted using ethanol (70:30, *w*/*v*) for 24 h at room temperature.

### 3.5. Qualitative and Quantitative Analysis of Rutin Using High-Performance Liquid Chromatography

Briefly, 0.3 g of the lyophilized sample was dissolved in 3 mL of EtOH (60% aqueous solution). The content of rutin was monitored using a diode-array detector (DAD, 2998, Water Corporation, Milford, MA, USA). Analytical detection was conducted from 200 to 600 nm. Rutin was detected at 354 nm. Analytical separations were carried out on a LiChroCART Purospher^®^ STAR RP-18 endcapped (250 × 4.6 mm, 5 µm particle size) column along with a guard column, Purospher STAR RP 18e 4.0 × 4.0 mm 5 µm (Merck KgaA, Darmstadt, Germany). The temperature of the column was 25 °C. Gradient elution was performed with a mobile phase consisting of 0.1% aqueous solution of formic acid (solvent A) and acetonitrile (solvent B), with the flow rate set to 1 mL/min. A linear gradient profile was applied with the following proportions of solvent A: initial—100%, 30 min—85%, 50 min—50%, 55 min—0%, 60 min—100%. All the samples were filtered through a 0.45-mm polyvinylidene fluoride (PVDF) syringe filter (Millipore, Burlington, MA, USA) before injection. To quantify rutine in the samples, a calibration curve was generated using an authentic rutin (SigmaAldrich, Burlington, MA, USA), standard. The obtained data were processed with the Waters Empower software (Waters Corporation, Milford, CT, USA).

### 3.6. Water Contact Angle Measurement

Static water contact angle measurements were performed using a goniometer (Firsttenangstroms (FTA32), ALV Technologies Pte Limited, Newark, CA, USA). Digital images of a 2 water droplet on the surface were captured and analyzed using the Fta32 Video software. Three different spots on the surface were tested for each sample and the average of the contact angle value was reported. A control sample was prepared without the use of a non-starch polysaccharide enzymes mixture.

### 3.7. Statistical Analysis

Mean values and standard deviations were calculated using MS Excel 2020 (Redmond, WA, USA). One-way analysis of the variance (ANOVA) for response surface were performed and calculated using Design-Expert 7.0.0 software (Stat-Ease Inc., Minneapolis, MN, USA). Moreover, an ANOVA along with the posthoc Tukey’s HSD test was employed for statistical analysis.

## 4. Conclusions

The optimization of common buckwheat extraction using a multienzymes mixture showed that the optimal conditions obtaining the highest hydrophilic extract yield (272 g), and total phenol content (324 mg GAE 100^−1^ g) were 24 h, 65 °C and cellulase activity of 8.6 CellG5 Units/mL. Temperature, time and enzyme activities play an essential role in extraction. These results showed the significant potential of improved excellent management of enzyme-assisted extraction for the development of higher added-value products, and green synthesis of various substances such as nanoparticles. Furthermore, hydrophilicity measurement using contact water angle was implemented for buckwheat multienzyme-assisted water extraction, which demonstrated that extraction time had an impact on hydrophilicity. Results are essential for developing novel functional foods due to growing consumer demand for plant-based food and food free from chemical additives.

## Figures and Tables

**Table 1:** Detailed experimental extraction runs and results on yield and total phenolic content.

Variation Levels and Runs	Variables			Liquid Yield, g	TPC mg GAE/100 g
	Time, hours	Temperature, °C	NSP, mL		
Low level	2	60	0.10		
Medium level	13	70	0.55		
High level	24	80	1		
1	24	70	0.55	266	300
2	13	70	1.00	251	283
3	13	70	0.55	251	350
4	13	80	0.55	262	228
5	2	80	1.00	250	259
6	13	70	0.55	250	294
7	13	70	0.55	265	317
8	13	70	0.55	262	299
9	24	60	1.00	289	289
10	24	80	1.00	214	294
11	2	80	0.10	246	260
12	2	60	1.00	267	282
13	2	60	0.10	246	309
14	2	70	0.55	268	336
15	13	60	0.55	268	249
16	13	70	0.10	254	294
17	13	70	0.55	262	262
18	24	80	0.10	196	256
19	13	70	0.55	260	299
20	24	60	0.10	277	262

**Table 2:** Water contact angle measurement results of different optimization run samples.

Sample Name	Fraction	Hot Water Extraction Time, hours	Cellulase Activity CellG5 Units/mL	Water Contact Angle, ° (degree)
Control	Solid	24	-	39.0 ± 1.3
	Water	24	-	47.8 ± 5.4
1	Solid	13	8	35.6 ± 6.1
	Water	13	8	57.5 ± 3.6
2	Solid	13	4	38.1 ± 0.3
	Water	13	4	57.4 ± 9.0
3	Solid	24	4	35.7 ± 5.0
	Water	24	4	50.7 ± 4.0

**Table 3:** Nutritional declaration of common buckwheat flours (g/100 g).

Energy value (kcal/kJ)	353/1497
Fat, g	3.0
Saturated fat, g	0.7
Carbohydrates, g	68.8
Sugars, g	1.3
Proteins, g	12.6
Salt, g	0

## Data Availability

The data presented in this study are available in the article.
